# 

**Published:** 1949-04

**Authors:** 


### Local Medical Notes

**The Thirty-Eighth Long Fox Memorial Lecture** will be given in the University of Bristol, on Tuesday, 18th October, 1949, by Professor J. E. HARRIS, M.A., Ph.D., on "**The use of Tracer Elements in Biology and Medicine.**"

---

VISIT OF PROFESSOR GREY TURNER TO BRISTOL

Professor G. Grey Turner honoured the University and Teaching Hospital by a visit from 21st February to 25th February, 1949. His name has long been a household word in British Surgery and many generation of students, both in Newcastle and at the Postgraduate Medical School in London, have benefited from his teaching. He was a great traveller and his discourses always reflected the benefits to be obtained from visits, official or unofficial, to other active centres of surgery, and in consequence, visits to see him at work either in Newcastle or London always provided a rich reward.

During his week in Bristol he undertook an active part in the work of the Department of Surgery. He attended the weekly conference in the Pathological Department and a Surgical Pathological Conference. He taught in the wards, he taught in the outpatient department, and he demonstrated his skill in the operating theatre by performing a difficult sub-total gastrectomy for a high gastric ulcer by the Bilroth I method. In addition he attended ward rounds in the Thoracic Surgical and Neurosurgical Departments at Frenchay Hospital.

But perhaps the high spots of the visit were two lectures which he gave to enthusiastic audiences of staff and students. The first of these lectures dealt with some long-term results of the treatment of malignant disease and was illustrated by remarkable photographs of patients and pathological specimens, some of them dating from the early years of the century. This lecture finished with a note on the work of the Research Defence Society and its importance in medical practice. His second lecture described his achievements in transplantation of the ureters, mainly in patients with congenital abnormalities. He showed by the personal way in which he discussed them the human feeling which he has towards all his patients, for many of these have been his friends from childhood until they had brought up happy families themselves. In this lecture he turned to the prominent leaders of British Surgery since the days of Hunter, and paid particular tribute to Greig Smith and Hey Groves of Bristol, especially mentioning the debt which he owed to the former for his textbook.

Professor Grey Turner qualified in the University of Durham more than half a century ago and he speaks with a life-long experience of active surgery. His visit to Bristol will long be remembered by all who had the opportunity of meeting him and hearing his teaching. In the future when his name comes up amongst the great surgeons of the first half of the twentieth century, people will look back with satisfaction and think " I remember the occasion when Grey Turner visited Bristol ", and cherish the memory of seeing this great man at work.

---

### DEGREES AND DIPLOMAS.

**University of Bristol.**—M.D.—J. B. Brierley.

D.M.R.D.—C. H. Kitchen.

M.B., CH.B.—*Second Examination*—F. J. Appelbe, W. M. Baggott, K. J. Barker, B. G. Bennett, A. R. Bollen, Ann E. Booker, J. C. Causton, Anne W. Christie, Jean M. Claremont, N. J. Cobb, B. J. F. Coles. B. Colston, P. A. Crook, W. J. P. Duff, K. W. Edmondson, K. J. R. Fawcett, H. G. Gage, E. C. Gawthorn, D. J. Gear, B. Ginsberg, W. E. L. Gordon, E. P. Hamblett, G. L. Higgins, Elizabeth M. James, J. W, Jenkins, A. F. Jordan, Janet H. Keevil, B. Livesey, G. P. Lowther, A. Moore, A. B. Otlet, I. W. Palmer, T. F. J. Parker, Ann C. Parry, Barbara M. Podger, M. N. Prichard, Alison W. Prowse, T. D. Richards, N. G. Sanerkin, R. A. Sellwood, P. H. Sherwood, H. S. Smith, J. E. Tovey, H. A. P. Walker, D. E. Worsley.

**Royal College of Physicians.**—M.R.C.P.—M. J. Greenberg.

**Royal College of Surgeons.**—D.L.O.—G. G. Allan, T. C. Forte.

**Royal College of Obstetricians and Gynaecologists.**—M.R.C.O.G. :— J. M. Duncan.

